# A qualitative study exploring pregnant women’s weight-related attitudes and beliefs in UK: the BLOOM study

**DOI:** 10.1186/s12884-015-0522-3

**Published:** 2015-04-22

**Authors:** Uma Padmanabhan, Carolyn D Summerbell, Nicola Heslehurst

**Affiliations:** Health and Social Care Research Institute, School of Health and Social Care, Teesside University, Teesside, TS1 3BA UK; School of Medicine, Pharmacy and Health, Durham University, Queen’s Campus, Stockton-on-Tees, TS17 6BH UK; Institute of Health & Society, Newcastle University, Newcastle-upon-Tyne, NE2 4AX UK

**Keywords:** Pregnancy, Gestational weight gain, Diet, Physical activity, Weight management, Qualitative

## Abstract

**Background:**

There is little information on the individual cognitive, perceptual and psychosocial factors that influence the lifestyle behaviours of pregnant women. This study explored pregnant women’s weight-related attitudes and beliefs during pregnancy.

**Methods:**

Nineteen pregnant women with different pre-pregnancy BMIs and in their third trimester were purposefully sampled for face-to-face interviews. Topics covered included lifestyles, sources of information, feelings about their bodies, and level of control over themselves and their bodies. Systematic thematic content analysis was used to identify recurrent themes.

**Results:**

Women perceived their bodies as fragmented into ‘my pregnancy’ (the bump) and ‘me’ (rest of my body). This fragmentation was the key driver of their weight-related attitudes and beliefs and influenced their dietary and physical activity behaviours. Consuming healthy foods was necessary for ‘my pregnancy’ to provide the ideal gestational environment. Simultaneously, pregnancy was perceived as a time to relax previously set rigid rules around diet and physical activity, allowing women to consume unhealthy foods and lead sedentary lifestyles. Women faced emotional conflicts between limiting weight gain for ‘me’, and being perceived as acting morally by gaining enough weight for ‘*baby*’. Although ‘*bump’* related weight gain was acceptable, weight gain in other parts of their body was viewed negatively and implied lack of self-control. Conflict was often alleviated, and weight-related behaviours validated, by seeking practical and reputable information for weight management. Women felt that their midwives provided detailed information on what they should not do during pregnancy, but were rarely given information about what they should do in relation to diet and physical activity for weight management. Consequently, women often used information from a variety of sources which they filtered using ‘common sense’.

**Conclusions:**

This study has identified that a central concept to pregnant women’s diet and physical activity beliefs during pregnancy is the fragmentation of self into ‘me’ and ‘my pregnancy’. This fragmentation influenced beliefs about diet and physical activity, and control and acceptability of gestation weight gain on different parts of the body. Future interventions and antenatal care should take this fragmentation into consideration when providing pregnant women with advice, information and support relating to their diet and physical activity behaviours.

## Background

Excess gestational weight gain can increase risks to women during pregnancy, labour, and postnatally, including pre-eclampsia, gestational diabetes, caesarean section, instrumental delivery, and postpartum weight retention [1]. Additional risks to the infant include pre-term delivery, macrosomia and risk of early childhood obesity [1-5]. To maintain optimal maternal and fetal health during pregnancy, the Institute of Medicine (IOM) in the United States of America (USA) has published recommended gestational weight gain ranges according to early pregnancy body mass index (BMI) [6]. Although there exists a wide range of gestational weight gain guidelines by different countries throughout the world, almost half of the countries had similar IOM guidelines [7]. The UK guidelines for weight management during pregnancy [8] recommends that women’s height and weight should be measured as early as possible in pregnancy, and that this measurement should be used to plan her subsequent care. However, these guidelines do not include specific gestational weight gain recommendations, and routine weight monitoring is not recommended unless there is a clinical need. As gestational weight gain trends in USA and Europe suggest that less than one-third of pregnant women gain weight within the IOM guidelines [9-13], the National Institute for Clinical Excellence (NICE) in the UK has specifically recommended that further research be carried out in UK populations relating to gestational weight gain [8].

Numerous complex interventions have been designed to prevent excess gestational weight gain, consisting of interacting components including nutritional and physical activity counselling [14,15], and motivational talks such as individual counselling sessions on weight control and supportive talks using motivational interviewing [16,17]. The effectiveness of these interventions on gestational weight gain are inconsistent with some reviews reporting no clear evidence of effect, and others reporting some reductions in gestational weight gain [3,18-21]. Limitations in the interventions’ effectiveness have been attributed to poor study design, lack of power, lack of consistency in the behaviour being targeted (e.g. either physical activity or diet), and not tackling behaviour change or motivation [20].

Although habits play an important role in lifestyle behaviours, ‘motherhood’ is perceived as a time of transition into a new role, bringing about changes in health concerns, expectations of self-image and weight gain, and responsibilities [22]. It also has deep-rooted socio-cultural roots that embody certain stereotypes of ‘good’ versus ‘bad’ mothering behaviours [23]. Moreover, the legally elevated status of the fetus as an individual entity, separate from the mother, has resulted in focussing public attention on the behaviours of pregnant women and heightened the vigilance expected of them [24]. As a result, lifestyle behaviours during the transition to motherhood are bound by the ideologies of appropriate mothering behaviours, which are in turn reinforced socially and culturally [25]. Consequently, pregnant women also re-evaluate their behaviours and their relationship with society during transitional phases, from pre-pregnancy, to pregnancy, and postnatal [26]. As transitions can often lead to changes in personal and social identities or status which provide the impetus to re-evaluate behaviours, it provides an opportune time for changing behaviours [27].

Due to difficulties in altering diet and physical activity behaviours directly, influencing the ‘choices’ that people make is an appropriate strategy for the modification of behaviours [28]. While conventional beliefs can limit pregnant women’s choices in terms of ‘what’, ‘when’ and ‘how’ to eat, they can also sanction consumption behaviours; e.g. eating for two, or giving in to one’s cravings [26]. Pregnant women’s food choices are therefore intimately linked to social-cognitive factors influenced and shaped by the environments they encounter, as well as from previous transitional experiences [29]. Moreover, physiological changes associated with pregnancy also influence food choices, such as cravings, nausea and food aversions [30]. A systematic review [31] that examined the determinants of physical activity during pregnancy found that intention to exercise, self-efficacy, and barriers such as lack of time, tiredness or physical limitations were strong predictors of exercise. However more information is required on the motivational factors that impact on pregnant women’s self-efficacy or enable them to overcome the barriers to exercise during pregnancy [31,32]. Studies examining gestational weight gain have also reported that cognitive factors such as motivation and body image concerns were predictors of gestational weight gain [33]. Hill *et al.* [33] also argued that as people who have lower body image were more likely to utilise negative coping skills during pregnancy, there could be a relationship between body concerns and lack of intrinsic motivation to engage in physical activity or healthy eating behaviours.

As the mechanisms that result in behaviour change in this context are not very clear, having in-depth information on the individual cognitive, perceptual and psychosocial factors that influence lifestyle behaviours during pregnancy could help guide and inform action strategies when counselling pregnant women or developing interventions related to diet and physical activity. As dietary and physical activity behaviours are a function of the salient beliefs and attitudes that people hold relevant to them, this study aimed to examine pregnant women’s weight-related attitudes and beliefs (including the weight-related behaviours of diet and physical activity during pregnancy).

## Methods

An interpretive constructionist approach was used to elicit pregnant women’s weight-related attitudes and beliefs. This approach is an offshoot of two philosophies: phenomenology (the study of people’s experiences), and hermeneutics (the study of human existence through the interpretation of texts, verbal and non-verbal communication, and language) [34]. Using this approach allowed the exploration of pregnant women’s experiences within a socially constructed world, whilst allowing the researcher to be equally conscious of her own background and experiences.

The participant recruitment, data collection and analysis followed an iterative process. All the participants were previously recruited to a prospective quantitative longitudinal study which explored diet and physical activity behaviours during each trimester of pregnancy; the BLOOM study (Behaviour and Lifestyle Observation of Mothers). Women from a large maternity unit in North East England (South Tees National Health Service (NHS) Trust) were recruited to BLOOM using postal methods. Inclusion criteria for BLOOM were women aged over 16 years, singleton pregnancy within the first trimester, and able to read and write English.

**Figure 1:**
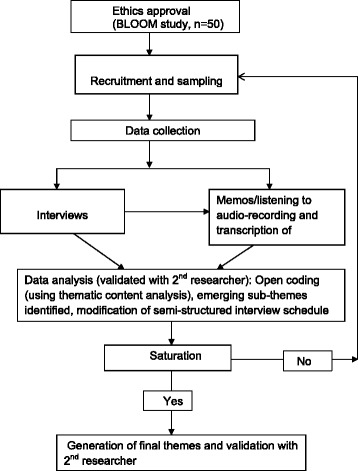
Flow chart of iterative methods of recruitment, data collection and thematic content analysis.

Women who were participating in the longitudinal study and were in their 3^rd^ trimester during the recruitment period were invited to take part in this qualitative study through invitation letters and participant information sheets. Convenience sampling was the initial sampling strategy, and women who were the first to respond were contacted and interviewed. This was followed by purposive sampling to ensure a well-representative sample in terms of gravidity, age, and educational status; this data was available to the researchers as it was self-reported by the participants at the start of the longitudinal study. Purposive sampling was carried out to attempt to explore a wide variation of perspectives so as to get greater insights into pregnant women’s attitudes and beliefs and also explore any differences in attitudes and beliefs as a result of these factors. Pre-pregnancy BMI and gestational weight gain (around 38^th^ week) data were only available to the researcher towards the end of the participant’s 3rd trimester and therefore could not be used to inform the purposive sampling during pregnancy. On completion of the interview, a £10 shopping voucher was provided. Sampling continued until theoretical saturation was confirmed (Figure 1).

### Data collection

All interviews were conducted face-to-face at a convenient venue suggested by the participants. The women consented to the interviews being audio-recorded. The initial topics covered in the semi-structured interview schedule explored changes in women’s routine practices from pre-pregnancy to during pregnancy and their feelings about their lifestyles and their bodies during pregnancy (Table 1). The interviewing technique was influenced by the responsive interviewing model. This is a dynamic iterative process with the intention of generating depth of understanding rather than breadth [35]. Prior to the interviews the women were aware that the researcher did not have a clinical background, and were asked to talk freely on any weight-related issue that was of interest to them. The interview schedule was mainly used to initiate discussions, and was flexible. Questions were allowed to develop naturally based on the information being provided by the participants. The prompt questions were utilised when necessary, to obtain depth, to ensure continuity, and to ensure similarity of topics covered among all the participants. All interviews were audio recorded and transcribed verbatim. A copy of the transcripts was also sent to the participants for verification and they were instructed to inform the researcher for any discrepancies.

**Table 1:** **Main topics discussed in the interviews before and after modifications**

**Interview schedule before modifications**	**Interview schedule after modifications**
Topic 1: Are there any changes in your dietary habits from before you became pregnant and now during this pregnancy? If so what are the changes?	Topic 1: What are your feelings about your lifestyle during your pregnancy?
Topic 2: Are there any changes in your physical activity from before you became pregnant and now during this pregnancy? If so what are the changes?	Topic 2: How do you feel about your body now compared to before you were pregnant?
Topic 3: Where do you normally obtain information about healthy eating and physical activity in pregnancy?	Topic 3: How much control do you feel that you have over yourself and your life during this pregnancy?
Topic 4: How do you feel about your body weight /image now compared to before you were pregnant?	

At the end of each interview, a brief memo was written up by the researcher outlining the most vivid facets of each interview. This included any key topics of discussion that were most obvious during the interview, the general feel of the interview, and the participant’s reactions to questions and emotional state during interviews. Repeated listening to the audio-recording of the interviews was used to expand the memos, and concurrently identify emerging themes. These themes were substantiated or disproved by thematic content analysis [36] and by comparing them with data from previous or subsequent interviews.

### Data analysis

Thematic content analysis, which is adapted from grounded theory, and content analysis was carried out by two researchers independently (UP and NH), using a systematic approach of immersion in the data, coding, and data interpretation into themes [36]. Each researcher independently analysed the data and identified categories. Independent analyses were compared to identify commonalities as well as differences, and grouped into interpretive themes. Independent analysis by a second researcher was used to increase the credibility of the interpretation of the findings, and offset researcher bias.

An initial thematic content analysis of the first four interviews was carried out to explore whether any changes to the interview prompts were required to gain more in-depth responses, and to follow up on any early emerging themes. Reflexivity is a process that allows the researcher to reflect on self-bias, preferences, and theoretical predispositions [37]. The researcher had experiential and anecdotal information on behaviours during pregnancy, but did not want to enter the analysis with previously formed hypotheses. Therefore, rather than immerse oneself in substantive theories around body weight, a preliminary reading of the literature on pregnancy-related behaviours was limited to inform research questions. There was limited evidence on pregnant women’s weight-related attitudes and beliefs, however literature on self-identity and body image during pregnancy, socio-cultural representations of the female body, risk and responsibilities during pregnancy, and the medicalisation of pregnancy were plentiful [24,38-45]. After the preliminary analysis of the data, substantive theories around body weight in women were accessed to direct subsequent interviews in order to increase the depth of findings and ensure that they were theoretically grounded.

The initial interview schedule was therefore modified to pursue emerging themes, and to generate increased depth in response (Table 1). The whole corpus of data was analysed using the same process [36]. This literature therefore provided the context and evidence for the construction and shaping of the major three themes presented in this study.

The analysis is supported by participant quotes, and all participants were allocated pseudonyms to maintain anonymity. To represent the voices of the participants all slang words, grammatical imperfections, and emotional cues such as laughter or pauses have been preserved. Brief pauses are indicated with three dots and longer pauses have additional dots. When the participants digressed from the topic, the unrelated conversations have been deleted and these are indicated with square brackets […]. When additional information is required to provide context to the quote, this is marked within square brackets (e.g. [diet]).

Approvals were obtained from Teesside University’s School of Health and Social Care Research Ethics Committee (REC), County Durham & Tees Valley 2 NHS REC, and South Tees NHS Trust Research and Development committee. All the participants provided written informed consent prior to their interview.

## Results

All participants were residents of Tees Valley, situated in North-East England (Table 2). The age of the participants ranged from 19–38 years, gravidity from 0–5, BMI groups included recommended (18.5 – 24.9 kg.m^−2^), overweight (25.0 – 29.9 kg.m^−2^) and obese (>30.0 kg.m^−2^). The ethnicity of all except one woman was White. With the exception of one participant who was interviewed at her place of work, all other women were interviewed in their own homes. Analysis of the data identified three overarching themes and sub-themes (Table 3).

**Table 2:** **Individual participant characteristics**

**Participant Pseudonym**	**Age**	**Educational level**	**Booking BMI status (WHO guidelines)**	**Gravidity**
Amy	30	A-levels	Recommended category	Second pregnancy
Trudy	27	A-levels	Overweight category	Second pregnancy
Katie	37	A-levels	Obese category	Fifth pregnancy
Michelle	28	GCSE	Overweight category	Second pregnancy
Moira	27	A-levels	Recommended category	Second pregnancy
Brenda	28	GCSE	Not available	Third pregnancy
Alex	35	GCSE	Overweight category	Second pregnancy
Pam	37	A levels	Recommended category	First pregnancy
Jean	27	GCSE	Overweight category	Second pregnancy
Sam	36	Graduate	Recommended category	Second pregnancy
Jenny	29	GCSE	Overweight category	First pregnancy
Holly	40	Others	Obese category	Sixth pregnancy
Sally	23	Others	Recommended category	First pregnancy
May	27	GCSE	Recommended category	First pregnancy
Daisy	19	Others	Overweight category	First pregnancy
Anne	33	A levels	Recommended category	First pregnancy
Mary	31	Post-graduate	Recommended category	First pregnancy
Rose	25	A levels	Overweight category	First pregnancy
Debra	38	Others	Recommended category	Second pregnancy

**Table 3:** **Themes and sub-themes**

**Theme 1: Fragmentation of the self –‘me’ and ‘my pregnancy’**	**Theme 2: Legitimising behaviours**	**Theme 3: Body and behaviour surveillance**
**a) ‘me’**	**a) Validation of behaviours**	**a) Behaviour surveillance**
-- Mothering norms		
-- Weight-related conflicts		
-- Locus of control (internal)		
**b) ‘my pregnancy’**	**b) Justifications**	**b) Body surveillance**
-- Minimising conflicts	-- Changes: acceptance	-- Assessing weight gain
-- Locus of control (external)	-- Justifying diet	-- Feelings about weight gain
	-- Barriers to PA	

### Theme 1: Fragmentation of the self – ‘me’ and ‘my pregnancy’

This theme illustrates the fragmented way in which the pregnant women in this study perceived their bodies. One fragment was ‘my pregnancy’ or the ‘bump’ which referred to the protruding stomach encompassing the fetus and the other products of conception. The rest of their body minus the bump was ‘me’. Women’s comments throughout the interview suggested that they perceived the bump as sometimes being a part of themselves, and also apart from their selves. When women talked about their bodies, they felt confident and knowledgeable about ‘me’, but were often less so about the bump. This theme dominated all of the interviews, and is a central concept underpinning the other themes.

*“I’ve been … just knowing my own body, umm… I wouldn’t know with my stomach* [bump], *but with my legs and stuff* [me] *I know that I have put too much* [weight] *on”* (May, 1^st^ pregnancy, Recommended BMI).

#### Mothering norms (‘me’)

Due to this fragmentation, the foremost feeling that prevailed throughout the interviews was the sense of responsibility in providing the ideal gestational environment for ‘my pregnancy’. Women repeatedly discussed the virtues of focussing attention on the fetus rather than being perceived as self-absorbed with their body weight.

*“It’s what you give your baby that is more important rather than what you think of yourself isn’t it?”* (Jean, 2^nd^ pregnancy, Overweight BMI)

While this focus illustrated a moralistic perspective, it also exemplified good mothering norms as consuming a healthy diet was essential for providing the fetus with all the nutrients required for its growth and well-being.

*“I would like to think that I have done everything possible to make sure that the baby is healthy and when it is born, has a healthy weight and is not being deprived of anything”* (Rose, 1^st^ pregnancy, Overweight BMI)

Women often protected their own self-image by pointing out how they adhered to norms even if other pregnant women did not. Women equated fatness with inactivity and overeating, and terms such as “*greedy*”, *“pig”* “*lazy”* and *“fat”* were used to describe excessive weight gain in a derogatory and disapproving manner, also alluding to women with a lack of self-control.

*“I think if I put on like five stone on quickly or something ridiculous, then I would have known that I never stopped eating rubbish, then I would feel that was not baby weight but me being greedy”* (Anne, 1^st^ pregnancy, Recommended BMI)

*“I don’t think you should just be lazy and use pregnancy to be lazy just because you are pregnant. Okay there are things you are not allowed to do, I can’t move anything heavy, but it doesn’t mean I can sit all day and be lazy* (Sally, 1^st^ pregnancy, Recommended BMI)

When discussing physical activity, women felt that physical activity during pregnancy only benefitted ‘me’ by way of psychological and physiological benefit (e.g. helped them relax, maintained fitness levels, and enabled an easy labour). Conversely, they felt that certain forms of physical activity could put ‘my pregnancy’ under severe risk. Therefore, the nutritional benefits of diet to ‘my pregnancy’ were prioritised over the benefits of physical activity to ‘me’. Additionally, the avoidance of physical activity risk to ‘my pregnancy’ was prioritised over the potential benefits to ‘me’.

*“Obviously it’s* [diet] *more important during pregnancy because you’ve got another person to think about, not just yourself”* (Michelle, 2^nd^ pregnancy, Overweight BMI)

*“I think that the not eating too much junk was a higher priority than going swimming or whatever, or you know just doing some type of physical activity, even though I know they go hand in hand”* (Trudy, 2^nd^ pregnancy, Overweight BMI)

#### Weight-related conflicts (‘me’)

Fragmentation of the self was further evident when women discussed their feelings about their growing body. Women accepted that weight gain was integral to their pregnancy, but simultaneously wanted to limit their weight gain to their bump (‘my pregnancy’). For many women this introduced varying degrees of weight-related conflicts.

*“I’ve got two sides to it, sometimes I get myself quite upset about the fact that I have put this two and a half stone-ish on, and obviously my arms are a bit bigger, my face is a bit bigger, my hips are a bit bigger, not that it ever bothers me, it’s not… at the end of the day there’s a baby in there, but… sometimes I do get myself quite upset by the weight I’ve put on…* […] *Had I had my own way I’d still like to stay quite slim and only have a bump”* (Michelle, 2^nd^ pregnancy, Overweight BMI)

*“I’m partly satisfied, […] honestly if I were to tell you that I am happy with my size, that I’m one hundred percent satisfied that won’t be true, as I want to be a little bit slimmer (laughs), but that’s impossible”* (Brenda, 3^rd^ pregnancy, BMI not available)

#### Locus of control – internal (‘me’)

Women’s comments also illustrated a sense of accountability and self-regulation of behaviours. Although the majority of women were unsure of the extent that their behaviours influenced weight gain in ‘my pregnancy’, many of them firmly believed that they could control the weight gain on ‘me’ by controlling their diet and physical activity. As a result, restricting the weight gain to the bump was viewed as a form of self-achievement.

*“Now because I only have a short time to go, I look at the scales and it’s a big achievement, and that’s brilliant, as what I have gained has been sufficient for the baby, but not to put on myself if you like, so I’m actually quite proud of myself* [laughs]” (Holly, 6^th^ pregnancy, Obese BMI)

*“I think it’s more because of my diet and everything and the way I am eating and I can actually see that you know it is working, not eating too much, I mean I’ve put weight on obviously on your breast I mean because of your breast milk and tummy because of the baby, but other than that I’ve not really put much on in other areas and still have a waist* [.*..*]* I look at myself in the mirror sometimes and think in the front I wouldn’t really look pregnancy until I turn to the side and see this big belly, so I think that helps you know, that you can physically see that I am in control”* (Jenny, 1^st^ pregnancy, Overweight BMI)

Even though women felt that the weight gain on ‘me’ could be controlled by their behaviours, not all lifestyle behaviours were equally easy to control. Diet was considered easier to control than physical activity, because of the numerous barriers to physical activity (discussed in Theme 2). However, the most difficult part of dietary self-control related to cravings. Women felt that cravings were very difficult to ignore, and they had to exert an immense amount of self-restraint.

*“I’ve got more control over my diet, because I’ve got more time to think about that, when I go out for lunch at work, and you can either choose to have something ridiculously fattening or go for a salad, so obviously I’ve got more control over my diet, but don’t have control over my exercise, because I simply don’t have the time for it”* (Michelle, 2^nd^ pregnancy, Overweight BMI)

#### Minimising conflicts (‘my pregnancy’)

Some women also described how some of their weight-related conflicts described earlier, were also minimised by focussing on the physiological changes associated with the bump. This focus allowed women to express their feelings of excitement and pleasure, and made them realise that some amount of weight gain was to be expected in order to have a healthy, normal-weight baby.

*“I’d rather get big myself and have a healthy baby rather than stay slim and end up having a little six pounder who wasn’t as healthy. So I tend to push all my feelings aside about my weight because I know that I’m going to end up having a healthy child because of it. So I can worry about myself after I gave birth”* (Michelle, 2^nd^ pregnancy, Overweight BMI)

*“I was weighed today by the midwife and I said, ohh have I put on?, but that doesn’t bother me because I thought I’ve never had a weight difference, I’ve always been a steady weight before I was pregnant, so I can feel I’ve put weight on, but it isn’t physically bothering me because obviously you know that it is a growing baby and not me”* (Anne, 1^st^ pregnancy, Recommended BMI)

Women who had varying degrees of weight-related conflict however felt that even though weight gain was acceptable on ‘me’ during pregnancy, weight retention after pregnancy was not. Therefore they asserted their need to re-gain control over their body after pregnancy by increasing their physical activity and taking control of their diet.

*“I’m just seeing it as a temporary kind of measure that’s going to… in 5–6 weeks hopefully things are going to get better and um… as soon as the baby is here, you know start getting back on track and I don’t think there is any point in sitting and getting upset about the things that I can’t do or that I want to do, you know it’s my choice to do this, so I’ve just got to … 9 months is not the end of the world”* (Debra, 2^nd^ pregnancy, Recommended BMI)

#### Locus of control - external (‘my pregnancy’)

Although women felt they could control the weight gain on ‘me’ through their behaviours, they were unsure whether the weight gain in the bump was influenced by their dietary and activity behaviours. As a result even women who felt they were in control of their weight on ‘me’ were not sure they had the same control over the weight gain in the ‘bump’.

*“Partial control, you know I mean, you can control to a certain degree how much weight you’ve gained… you know just by controlling the amount of food that you eat, umm you know you’ve got the other bit as well… there’s the extra blood and placenta and fluid and actual baby and those things you can’t control how much they weigh* (Trudy, 2^nd^ pregnancy, Overweight BMI)

*“I think there’s a level of control over most things, I think the only thing I have no control over is the pregnancy* [bump] *itself really. The whole process has to just take its course, doesn’t it? But I think that I do have a level of control over just about everything else* [weight gain on me]*”* (Moira, 2^nd^ pregnancy, Recommended BMI)

### Theme 2: Legitimising behaviours

When the women in this study were questioned on their feelings about their lifestyles during pregnancy, there were expressions of doubt about whether they were doing everything “right”. The origin of these doubts could be partly attributed to the type of diet and activity-related information they received from their midwife. This information was perceived as “*vague*”, *“lacking depth”*, *“insufficient”* and focussed on restrictions during pregnancy rather than proactive information.

*“I mean you know you get like a leaflet on foods to avoid, but you know there isn’t really that much information about you know well what you can eat… it’s more of a don’t do this and don’t do the other, so that I know what I’m not supposed to do, so surely what I’m allowed to do out-weighs the things that I’m not supposed to do?”* (Trudy, 2^nd^ pregnancy, Overweight BMI)

Women felt that midwives assumed they already had the requisite knowledge about diet and activity, or had the necessary skills to research the topic themselves. On the contrary, women did not always feel confident that they had the requisite expertise in managing their weight gain. Moreover, women preferred being given verbal advice from their midwife rather than assimilating all the written information provided.

*“I think rather than just giving people booklets and things to go and read themselves, maybe they* [midwives] *should have some one-to-one sessions where we are actually told this is what we should be doing,* [*…*] *so just to assume that somebody will do their own research, isn’t really a good idea, so I think that they* [midwives] *do need to be more direct, rather than just assume they* [pregnant women] *can go away and use a computer”* (Mary, 1^st^ pregnancy, Recommended BMI)

#### Validation of behaviours

Women in this study validated the changes to their behaviours: including both positive and negative changes. While both nulliparas and multiparas wanted to validate their behaviours, their reasoning for validating it varied. Nulliparas did not know what to expect, and the vast quantity of diverse information made it imperative for them to research the topic completely. On the other hand, even though multiparas were more experienced, the changing nature of information and availability of up-to-date information made it imperative to “*keep on top of the information”*.

As much of the information available was considered *“non-reputable”* it was filtered using their own innate awareness, referred to as “*common sense*”, or based on their previous experiences in managing their weight gain either during or before pregnancy. They therefore routinely engaged in reflective practices to distinguish between acceptable and non-acceptable dietary and physical activity behaviours, and to separate myths from facts.

*“I remember when I was a little girl and my dad would sit with that slice of bread and dripping smothered in salt. That’s how it was then, that’s what you would eat but as the years have gone on and there have been more studies into food, and what’s good for you and not good, it changes over the years, so you are more aware as the years go on”* (Holly, 6^th^ pregnancy, Obese BMI)

Women also expressed concerns about the safety of physical activity during pregnancy to their baby. They were especially worried as most of the exercises recommended such as walking or swimming needed conducive weather. Most women therefore pondered the benefits of indoor exercises and questioned the absence of any recommended indoor-exercises which could minimise sedentary behaviours. Moreover, there was some reluctance among women to attend exercise classes if they were not recommended by their midwife due to uncertainties about the expertise of the trainers, and preferred classes run by midwives or trained professionals. However, some women were having difficulties in getting this type of information from their midwives.

*“It’s hard when you’re pregnant to find an exercise that is suitable for you. I mean they do say do your walk, … a lot of that is like weather permitted, and the number of aqua natal classes, there’s only the one I’m aware of, I know that my local pool started doing their own, but I’m not sure who is doing it because it’s not been advertised through the midwife, but been advertised through their own sources”* (Debra, 2^nd^ pregnancy, Recommended BMI)

#### Justifications

From the initial interviews it also became apparent that when women talked about their lifestyle behaviours, they automatically provided an explanation for any change without any prompting. Behaviours that were defined as ‘deviant’ by the women themselves were routinely justified. Justifications are accounts in which the person accepts responsibility for the action, but denies any pejorative quality associated with it [46]. Therefore in justifying these deviant practices the women absolved themselves of any associated negative qualities. The types of justifications women offered are summarised in Tables 4 and 5. Justifications connected to dietary behaviours were associated with reasons for consuming foods women perceived as unhealthy and also illustrate the fragmentation as they were for ‘me’ (Table 4). The justifications included relaxation of pre-pregnancy dietary rules (when unhealthy eating was socially unacceptable), pregnancy being a time when they could reward themselves with treats such as chocolates, blaming their consumption of unhealthy foods on cravings or *“what baby needs*”, justifying unhealthy choices by eating in moderation, and/or compensating for unhealthy food by justifying that they also consumed healthy alternatives, blaming unhealthy food choices on busy lifestyles and convenience. Whereas justifications connected to physical activity were related to barriers (Table 5) and leant towards both ‘me’ and ‘my pregnancy’ such as fear of harming the baby, physical limitations that prevented them from being active, constantly feeling tired as a result of the pregnancy, having no time, not having suitable facilities for physical activity or other environmental barriers.

**Table 4:** **Theme 2: Legitimising behaviours - Justifying diet**

**Justification presented**	**Supporting quotes**
**Relaxation of pre-pregnancy dietary rules:**	*“Well I, just tend to eat like sweet things every now and again which I would not have done before* [my pregnancy]*”* (Michelle, 2^nd^ pregnancy, Overweight BMI)
Women in this study described a relaxed attitude and increased temptation to consume foods that they classified as unhealthy during pregnancy. These discussions also implied that rules were relaxed to assuage their own needs rather than the need to provide an ideal gestational environment. i.e. for ‘me’ rather than for ‘my pregnancy’.	
**Treats:**	*“Sometimes you do just fancy a big bar of a chocolate, and it’s an excuse to pig out because I am pregnant, now I will have a little treat”* (Rose, 1^st^ pregnancy, Overweight BMI)
Eating foods purely for hedonic reasons, or as treats, was usually not acceptable to women prior to pregnancy. However, pregnancy allowed women to ‘indulge’, thereby legitimising treats.	
**Cravings:**	*“Now I think my body is wanting me to eat this and maybe the baby needs a bit of calcium with the cheese or a bit of sugar from the sweet things”* (Mary, 1^st^ pregnancy, Recommended BMI)
Women craved unhealthy foods which they felt were easily resisted when not pregnant. Cravings were perceived as physiological process over which the women had no control, or as the body’s or fetus’ need for particular nutrients. This perspective on cravings justified the consumption of previously restricted unhealthy foods.	
**Eating in moderation:**	*“I had more control* [over diet] *before I was pregnant whereas now I think well you know a little bit of what you fancy in moderation you can have, if it makes sense”* (Sam, 2^nd^ pregnancy, Recommended BMI)
Even though women felt that in pregnancy they could relax rigid rules, they were also aware of the consequential weight gain. Women reasoned that excess weight gain could be prevented by eating in moderation.	
**Compensation:**	*“I eat quite a lot of salad but I also eat quite a lot of chips and I know that chips are not healthy but I like them (laughs), in my mum’s house we eat a lot of veg and fruits, so I thought that was just enough really”*(Sally, 1^st^ pregnancy, Recommended BMI)
Women felt that consumption of some unhealthy food would not harm their baby as they would be provided with adequate nutrients from healthy foods regularly consumed, regardless of their consumption of unhealthy foods.	
**Busy lifestyles:**	*“We tend to eat a lot of convenience food because I’m working full time and more things like fish fingers, chicken nuggets* […] *its always just whatever is in the freezer type of things”* (Alex, 2^nd^ pregnancy, Overweight BMI)
Often women juggled work and family commitments during pregnancy. Even though they wanted to avoid eating unhealthy foods, often constraints in the way of time, feeling tired, and a lack of motivation justified their consumption of unhealthy foods.	
**Foods easily available and or addictive:**	“*I eat a lot better when I’m at work,* […] *I take my breakfast, my lunch and my tea, and there’s always fruit in, whereas at home I think you’re more…it’s easier just to go to the biscuit jar and get a biscuit”* (Sam, 2^nd^ pregnancy, Recommended BMI)
Snacking on unhealthy foods was justified on the grounds that it was easily available and hard to resist. Working women felt especially vulnerable as they tended to snack more on unhealthy foods during their maternity leave.

**Table 5:** **Theme 2: Legitimising behaviours - Justifying physical activity**

**Justification presented**	**Supporting quotes**
**Fear of harming baby**	“*I probably would have liked to have done some more activity, if the truth be known, … probably thinking I didn’t want to harm the baby which is completely stupid and I should have just got on with it, but I think… if I don’t do any exercise for 9 months, nothing can harm the baby”* (Pam, 1^st^ pregnancy, Recommended BMI)
When women had doubts about the safety of activities on their baby, they discontinued or reduced the intensity of certain regularly performed activities.	
**Physical limitations**	*“At the moment it is not a long walk to work, and by the time I get there I am a bit out of breath and stuff and I couldn’t walk more than that”* (Rose, 1^st^ pregnancy, Overweight BMI)
Women also reduced or stopped routine activities due to physical limitations making some activities more difficult. A few women also identified that their pregnancy related disorders, such as Symphysis Pubis Dysfunction (SPD) prevented their usual activities.	
**Tiredness**	*“I mean it is hard to explain why activity decreased* […] *when I come home I’m tired and I really don’t want to be getting organised and going swimming or going for a walk”* (Debra, 2^nd^ pregnancy, Recommended BMI)
Women reported juggling occupational work with family commitments, resulting in physical and mental tiredness.	
**Time limitations**	*“I do find it, I would find it difficult to go out and do a proper exercise routine, because I just physically don’t have the time I don’t get in much until 6pm and I leave the house at 6 am,”* (Michelle, 2^nd^ pregnancy, Overweight BMI)
Women felt that their work and family commitments left them little time to participate in structured physical activity.	
**Lack of services and costs**	*“I think there should be more free classes available for pregnant people* [*…*] *whereas when you are pregnant they don’t have free passes, and yet they have free passes for other people under 16 and over 65’s, although they are telling you that is good for you”* (May, 1^st^ pregnancy, Recommended BMI)
Some women identified a lack of subsidised or targeted services specifically tailored for pregnant women.	
**Environmental barriers**	*“It’s hard when you’re pregnant to find like an exercise that is suitable for you. I mean they do say do your walk, and obviously … a lot of that is like weather permitted”* (Debra, 2^nd^ pregnancy, Recommended BMI)
Bad weather conditions, lack of adequate transport and family support were restrictive to physical activity.	

### Theme 3: Body and Behaviour Surveillance

The pattern of response to multiple lines of questioning about lifestyle was usually weight-related among the women included in this study. This patterned response identified a relationship between behaviours, body weight, and satisfaction or dissatisfaction with the body. As discussed in theme 1, women experienced weight-related conflicts and these conflicts were also present within this theme. Women routinely assessed the weight gain by “*watching”* the amount and location of weight gain, which led to positive or negative feelings about their body. Since women believed that diet rather than activity was associated with weight gain, they also *“watched”* their dietary behaviours.

#### Assessing weight gain

As the majority of women did not receive weight gain advice from healthcare professionals, a variety of strategies were used to assess quantity and location of the weight gain. Clothing size was a significant method that both nulliparas and multiparas routinely used to assess weight gain. Comparing pregnant to pre-pregnant clothes size allowed women to conceptualise the degree of weight gain, and to visualise the location of the gained weight. Wearing the same size, albeit maternity clothes alluded to weight gained only on the ‘bump’. Alternatively, an increase in clothing size was interpreted as having gained excess weight on ‘me’.

*“Once I got pregnant, I’ve sort of stayed about…I would say about* [clothes size] *16, and I tend to buy clothes 16 still and just have to buy maternity sizes, so that they stretch with me.* […] *So I wouldn’t say I was you know weight wise I was unhappy sort of before …… but … because I’ve not really put a lot of weight on I’m quite happy”* (Jenny 1^st^ pregnancy, Overweight BMI)

#### Feelings about weight gain

Women’s self-assessment of their weight gain consequently led to either positive or negative feelings about their bodies. Location of the weight gain re-emphasised gendered identity by evoking feelings of *“sexiness”* and *“femininity”*. Many women welcomed the pregnancy increased or enhanced weight gain in certain parts of their body such as the breasts. Women who gained only bump weight, with minimal weight gain on ‘me’ were also happy and accepting of the weight gain. Multiparas, who gained less weight during this pregnancy compared to previous pregnancies, also had positive feelings about their bodies.

*“I like my boobs better* [laughs] *I’ve actually got some now… I didn’t have a big chest and now it’s gone up a couple of sizes and I quite like it. I just feel a bit more womanly which is quite strange, I didn’t expect them to grow like they have* [laughs] *I know they will probably go back to normal later”* (Anne, 1^st^ pregnancy, Recommended BMI)

Conversely, visible weight gain on ‘me’ led to negative feelings, and women found this weight gain *“upsetting”*. Both nulliparas and multiparas were therefore often wary of the changes taking place in their bodies, and sometimes mourned the loss of their former selves. Feelings were also influenced by the extent to which women perceived pregnancy as “*not normal”* in terms of external appearance (e.g. stretch marks), and from a functional perspective (e.g. physical limitations did not allow women to feel in control over their bodies). Consequently, these women wanted to regain control of their bodies and feel *“normal”* again.

*“I just can’t wait to get back to normal* [laughs] *you just get a bit fed up don’t you of having a big bump and especially like discomfort in your like pelvis and things umm… you just, you just want to wear your normal clothes and you look at everybody and they all look stick thin* [laughs] *compared to you”* (Sam, 2^nd^ pregnancy, Recommended BMI)

## Discussion

Fragmentation of the self into ‘me’ and ‘my pregnancy’ was instrumental in influencing pregnant women’s weight-related attitudes and beliefs in this study. Although other studies have identified feelings of fragmentation during pregnancy, its’ influence on women’s weight-related beliefs (and subsequent behaviours) is a novel concept.

Studies have described women’s perceptions of their bodies, and the women-fetus relationship as ‘*fluid*’ (sometimes merged or sometimes separate) [47], and as an ‘*invasion*’ [23]. Young [38] and Ussher [39] argued that the origin of these beliefs could be located in the contemporary view of pregnancy as a biomedical process rather than a natural process. They posited that this fragmentation is probably a reflection of the internalisation of the bio-medical norms governing pregnancy, leading women to believe that the fetus is not a part of their selves, but actually a separate entity with its’ own rules and regulations. Although this process completely de-authorises pregnant women of any control and authority over their pregnancy, it does not absolve them of their responsibilities to the fetus [41]. This phenomenon was clearly evident within the context of weight-related beliefs and attitudes in this study, as the women ascribed to the norm that the mother is morally responsible for mitigating any risks to the fetus. Dietary and physical activity attitudes and beliefs were underpinned by the prevailing medical and socio-cultural norms, and representations of ‘motherhood’ which were ascribed to and perpetuated during pregnancy. While women in this study were unsure of the extent to which their dietary or activity behaviours influenced weight gain on ‘my pregnancy’, most believed that their behaviours definitely influenced weight gain on ‘me’. Women equated fatness with inactivity and overeating, and although pregnancy weight was socially acceptable, contemporary pregnant ideals dictated that they limit the weight gain to the bump. Women’s beliefs that they were responsible for the amount and location of weight gain on ‘me’, while concurrently subscribing to their role as the caretaker of the fetus, perpetuated the self-regulation of behaviours. As a result, pregnant women tried to balance the weight gain in ‘my pregnancy’, and the weight gain on ‘me’.

Markens et al. [47] posited that pregnant women actively negotiate conflicting needs through dietary behaviours, as a means of balancing needs, desires and perceptions of overall health. In this study women discussed how their dietary and physical activity behaviours were not purely linked to their desire to provide an ideal gestational environment for ‘my pregnancy’, but paradoxically, pregnancy was also perceived as a time to relax the rigid rules set prior to pregnancy for ‘me’. The paradoxical nature of these beliefs suggested that although women believed that consuming healthy foods equated to a healthy and growing fetus, consuming unhealthy foods or leading sedentary lifestyles did not translate into increased risk. This belief was irrespective of women’s BMI status. However, not wanting to be perceived as behaving in a manner that compromised the ideal gestational environment, or wanting to be perceived as selfish and reckless women with little self-control, the women routinely validated and justified dietary and activity practices which they considered ‘deviant’. Consumption of unhealthy foods or increased sedentariness were acknowledged and concurrently justified, thereby negating any pejorative quality attached to them.

Wanting to limit the weight gain on ‘me’ also suggested that women still adhered to their pre-pregnancy ideals of body weight and shape. To some extent, the social acceptance of the weight gain during pregnancy tended to alleviate the pressures of non-conformity with cultural ideals of body size as well as any notions of lack of self-control. This allowed women to relax the rigid control over their dietary and physical activity behaviours during pregnancy. Mindful of the negative impact this relaxation could have on the weight gain on ‘me’, women sought reputable dietary and physical activity information. According to Lupton [48], the act of eating is a constant struggle for women; because it is perceived as a pleasurable act, as well as laden with irreversible consequences. When numerous options exist but little guidance on what and how one eats, individual choices are shaped by personal and social identities [30]. This was especially evident in this study, as the absence of reputable information resulted in women attempting to legitimise their behaviours.

The lack of reputable dietary and activity information has been similarly reported by other studies which examined lifestyle behaviours during pregnancy [26,49,50]. Gross and Pattison [26] argued that healthcare professionals’ merely advising pregnant women to eat ‘more healthily’ was not sufficient if women were unequipped with adequate knowledge to act on it. Similarly, the women in Weir et al*.’s* [50] study also reported that the foremost physical activity information they received from their midwives was to ‘carry on as usual’. Clarke et al. [49] also argued that the focus of most medical information and advice was usually about ‘more risky’ behaviours such as smoking or alcohol, with the specific aim of reducing or ceasing such behaviours. However, advice and information on other ‘less risky’ behaviours such as diet and exercise during pregnancy was less clear-cut [49]. This was similarly echoed in this study as women indicated that they received information on behaviours to avoid during pregnancy, whereas weight management information was limited. It is possible that this was a result of the lack of gestational weight gain guidelines within the current UK NICE guidelines for weight management during pregnancy [8] in the UK, at the time of study. Women were therefore receptive to information from non-medical sources, but relied on their own ‘*common sense’* to sift reputable from non-reputable information. As a result, diet was equated to an ideal gestational environment and a healthy baby (‘my pregnancy’), and therefore prioritised over physical activity which was perceived to only benefit the mother (‘me’) while potentially exposing the fetus to risk. These beliefs could be a major barrier to performing physical activity, as women did not feel compelled to be active during the pregnancy.

Women felt that their physical activity levels reduced during pregnancy, and many were unhappy with their level of activity. This was especially apparent when the weather was not conducive to the activities routinely recommended by their midwives. Women therefore deliberated on the value of home-based activity which minimised the effort to find the right clothes and childcare. Burke et al*.* [51] reviewed the effectiveness and benefits of home-based exercise routines in a non-pregnant population, performed with the support of healthcare professionals. They reported that increased contact with healthcare professionals led to improved exercise adherence, improved physiological and functional effectiveness, and increased quality of life. Considering women’s desire for, and the effectiveness of, home-based exercise, this could be a potential intervention for healthcare professionals to support women with physical activity. However, as reported in the meta-analysis [51], providing information alone is unlikely to be beneficial, and more proactive strategies are likely to be required.

### Strengths and limitations

Strength of this study lies in the timing of the interviews. Interviewing women in their third trimester allowed them to reflect on their lifestyles throughout their pregnancy. Moreover, taking a broad approach to women’s lifestyle and feelings about their body during pregnancy facilitated the exploration of the underpinning social and medical norms that influenced women’s weight-related attitudes and beliefs. The participants were also aware that the interviewer did not have any clinical background and was independent to the maternity services they were using. This could have resulted in women feeling less intimidated by the researcher, and potentially resulted in them disclosing information they would not have disclosed to a health professional or someone linked to their care team. The study population includes a diverse range of ages, BMI categories, and parity. This adds to the evidence base as recent qualitative studies with pregnant women relating to weight or weight-related behaviours focused primarily on women with a high BMI [50,52-55].

Participant characteristics, including socio-cultural norms and socio-economic status of some population subgroups, play a big role in developing behavioural attitudes and beliefs [56]. Studies examining socio-economic status and health outcomes have found the initiation and maintenance of healthy behaviours is dependent on individual-level resources such as knowledge, money, prestige, power and beneficial social connections [56], or a combination of contextual factors such as negative exposures [57] and the quality of the physical, social, and service environment [58,59]. One Irish study [60] reported that attitudes and beliefs vary across socio-economic status, and people of lower socio-economic status have stronger beliefs on the influence of chance on health and are also less likely to be health conscious. Moreover, lower socio-economic status was also associated with negative attitudes to nutrition, and the dietary profile of those with negative attitudes was poorer than those with a positive attitude. This study recruited women who were primarily white, and resided in one of the top 10 deprived regions in the UK and this could influence their health-related attitudes and beliefs as compared to those living in less deprived areas. Additionally, recruitment to the main BLOOM study was through postal methods and it is possible that the women in the sample pool from which the interview participants were drawn were more motivated and interested in diet and physical activity during pregnancy than the general pregnancy population. However, the purpose of qualitative research is not to generate generalizable results, rather to gain more depth of understanding.

## Conclusions

Findings in this study show that pregnant women tend to differentiate between acceptable and unacceptable gestational weight gain through a process of body fragmentation (acceptable weight gain in the ‘bump’ for baby, and unacceptable weight gain on other parts of their body minus the bump, i.e. ‘me’). Women associated the weight gain on ‘me’ with self-control, but paradoxically, pregnancy was also perceived to be a time when rigid controls over eating behaviours could be relaxed. As a result, supplementing healthy eating with hedonic needs for unhealthy foods was felt to be justified. As women reported sufficient information for maintaining the health of ‘my pregnancy’, but insufficient information for the regulation of weight gain on ‘me’, this paradoxical nature of their beliefs could lead to women gaining excess weight, as they may not have the required coping skills to recognise and manage the factors that increase their propensity to consume unhealthy foods. Moreover, it is possible that women who are unable to limit their weight gain to the ‘bump’ may perceive themselves as not having sufficient will-power resulting in negative self-image and or self-esteem.

### Implications for practice

Due to the benefits of healthy diets and physical activity [61], and the risks associated with excessive GWG, healthcare professionals have a providing sufficient information, support and encouragement for healthy lifestyles throughout pregnancy and to dispel diet and physical activity myths associated with pregnancy [8].

Women attached utmost importance to the information being received from their healthcare professionals, including midwives. As women felt that their behaviours were responsible for much of the gestational weight gain, healthcare professionals should use this as an opportune time to provide clear information on the nature of gestational weight gain as well as specify how much weight gain is required to ensure good pregnancy outcomes. Therefore having specific guidelines for gestational weight gain may be necessary in the UK to support this information provision. The paradoxical nature of beliefs wherein women believed that the weight gain on themselves could be controlled whilst also considering pregnancy as a time to relax rigid dietary rules, could lead to unwanted weigh gain. To prevent this, healthcare professionals could balance information on healthy eating for the pregnancy, with information on evidenced-based weight management strategies for pregnancy.

Healthcare professionals providing information for gestational weight management may require further education and support to recognise the contexts that influence pregnant women’s dietary and physical activity behaviours. A recent systematic review [62] has identified that health professionals face multiple and complex barriers to practice when trying to provide pregnant women with weight management support. Examples of barriers include a lack of knowledge of evidence-based recommendations for weight-management strategies, a lack of confidence in their behaviour change and communication skills, and beliefs that women will react negatively to weigh-related discussions which will have an impact on their relationship [62]. Therefore health professional education and training should take into consideration these barriers in addition to contexts which influence the pregnant women’s behaviours.

### Implications for future research

As this was a small localised study, the association between the fragmentation of the self and dietary and physical activity behaviours needs further exploration. Future studies could take into account socio-economic status and ethnicity to assess the extent of these feelings of fragmentation and any association with weight-related behaviours. Moreover, as midwives were perceived by women to be the experts during pregnancy, future studies could also document the experiences and challenges faced by midwives in the UK in providing suitable information on diet, physical activity and gestational weight gain.

Designs of future interventions should take into account this fragmentation, by being aware of the justifications women ascribe to which promote the consumption of unhealthy foods and demote the importance of physical activity during pregnancy. Interventions should therefore target both dietary and physical activity behaviours and be combined with counselling sessions to address any body weight conflicts that may influence behaviours, as well as to dispel myths related to weight gain, physical activity, and diet.

## Abbreviations

A levels: General certificate of Secondary Education Advanced level
BLOOM: Behaviour and Lifestyle Observation of Mothers
BMI: Body Mass Index
GCSE: General certificate of Secondary Education
IOM: Institute of Medicine
NHS: National Health Service
NICE: National Institute for Health and Care Excellence
REC: Research Ethics Committee
USA: United States of America
UK: United Kingdom
